# TMEM16A/ANO1: Current Strategies and Novel Drug Approaches for Cystic Fibrosis

**DOI:** 10.3390/cells10112867

**Published:** 2021-10-24

**Authors:** Christie Mitri, Himanshu Sharma, Harriet Corvol, Olivier Tabary

**Affiliations:** 1 Centre de Recherche Saint-Antoine, CRSA, Sorbonne Université, Inserm, 75012 Paris, France; christie.mitri@inserm.fr (C.M.); harriet.corvol@aphp.fr (H.C.); 2 Department of Biochemistry, All India Institute of Medical Sciences, Bathinda 151001, Punjab, India; himanshu.bcm@gmail.com; 3 Département de Pédiatrie Respiratoire, Hôpital Trousseau, AP-HP, 75012 Paris, France

**Keywords:** cystic fibrosis, anoctamin-1, calcium-activated chloride channel, CFTR-independent therapy

## Abstract

Cystic fibrosis (CF) is the most common of rare hereditary diseases in Caucasians, and it is estimated to affect 75,000 patients globally. CF is a complex disease due to the multiplicity of mutations found in the CF transmembrane conductance regulator (CFTR) gene causing the CFTR protein to become dysfunctional. Correctors and potentiators have demonstrated good clinical outcomes for patients with specific gene mutations; however, there are still patients for whom those treatments are not suitable and require alternative CFTR-independent strategies. Although CFTR is the main chloride channel in the lungs, others could, e.g., anoctamin-1 (ANO1 or TMEM16A), compensate for the deficiency of CFTR. This review summarizes the current knowledge on calcium-activated chloride channel (CaCC) ANO1 and presents ANO1 as an exciting target in CF.

## 1. Introduction

Cystic fibrosis (CF), an autosomal recessive genetic multiorgan disease, is caused by an absent or dysfunctional CF transmembrane conductance regulator (CFTR) channel that mainly mediates chloride anion transport across the apical membrane of epithelial cells. On the pulmonary level, CF leads to persistent pulmonary infections, chronic inflammation, and mucus plugging in the airways, causing irreversible lung damage. To date, more than 2000 different mutations in the CFTR gene have been identified [1]. A classification system groups mutations into six classes according to the functional consequences they generate on the CFTR protein: (1) class I: no functional CFTR protein; (2) class II: CFTR trafficking defects; (3) class III: defective channel gating; (4) class IV: decreased channel conductance; (5) class V: reduced synthesis of CFTR; (6) class VI: decreased CFTR stability at plasma membrane. Today, many symptomatic therapeutics (antibiotics, mucus thinners, bronchodilators, supplements to prevent malnutrition, etc.) are available to treat patients with CF, which has lengthened their life expectancy from 5 years in 1960 to over 50 years. New curative treatments aimed at rescuing CFTR dysfunctionality have emerged. There are four FDA-approved CFTR modulators developed by Vertex Pharmaceuticals (Kalydeco^®^, Orkambi^®^, Symdeko^®^, and the latest Trikafta^®^) [2], although the proven efficacy of these correctors and potentiators is limited only to particular mutations. There are still 15% of patients without any CFTR-directed therapeutics. Hence, there is an interest in finding an alternative strategy to treat patients with CF independently of CFTR mutations. Alternative ion channels have been suggested to bypass CFTR dysfunction [3,4], such as ENaC [5], the solute carrier 26A9 (SLC26A9) [6], and calcium-activated chloride channel (CaCCs), including anoctamin-1 (ANO1 or TMEM16A) [4]. Such approaches might be efficient therapies for all patients, regardless of their CF mutations.

## 2. Anoctamin-1

CaCCs were described for the first time in 1981 in *Rana pipiens* eggs [7] and then in *Xenopus laevis* oocytes [8]. Today, CaCCs have been identified in many cellular types (neurons, epithelial cells, smooth muscle cells, pancreatic cells, etc.) and have been shown to play essential roles in cellular functions [9,10,11]. Among CaCCs is the anoctamin family (ANO for anion channel and OCTA for their eight predicted transmembrane domains or TMEM16), consisting of 10 proteins (ANO1–10). ANOs might play an essential role in development due to their temporal and spatial differential expression in many developing tissues. We can separate ANOs into two groups: ANO1-2 and the rest. However, ANO2 has different biophysical characteristics to ANO1 and its expression is limited to the vomeronasal epithelium [12,13]. In 2008, ANO1 was identified as a CaCC by three independent research groups [14,15,16].

### 2.1. Structure of ANO1

**Figure 1:**
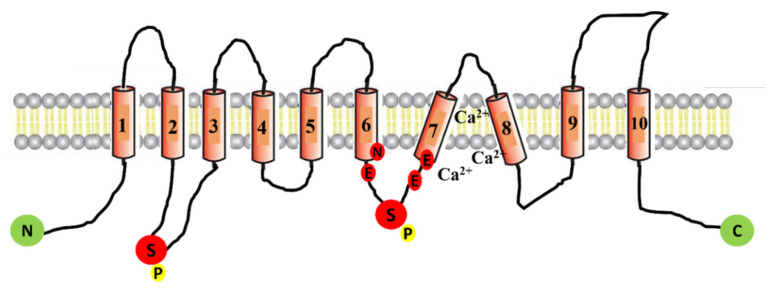
Schematic representation of ANO1 channel protein. The ANO1 channel protein consists of 10 transmembrane domains (TMD). The intracellular loop between TMD-7 and TMD-8 contains six amino acids (N650, E654, E702, E705, E734, and D738) that participate in forming the calcium-binding pockets of the channel. The depicted location of two phosphorylation sites (serine 471 and serine 673) is denoted with the letter P.

Since its discovery 11 years ago, many studies have been published describing the topological structure of ANO1. In 2011, a chemical crosslinking study performed by Sheridan et al. revealed that ANO1 exists as a dimer [17]. The dimeric structure of ANO1 was also confirmed by Fallah et al. using biochemical techniques such as native PAGE and chemical crosslinking [18]. Furthermore, Brunner et al. published the X-ray structure of calcium-activated ANO1 and showed that both subunits of ANO1 are symmetrical and have identical conformation [19]. However, two research groups recently proposed, on the basis of high-resolution single-particle cryoelectron microscopy, a common structure of ANO1 which is concurrent to the finding of earlier biochemical studies on ANO1 showing homodimers in the structure of anoctamins (Figure 1) [20,21]. Each subunit contains N- and C-terminal domains in this homodimer, a transmembrane unit consisting of 10 membrane-spanning α-helices, and extracellular components. Helices α1–α10 traverse the entire membrane, and a few of the helices are bent and tilted with respect to the plane [19]. Adjacent to the cavity, there are calcium-binding pockets, particularly α3–α7; using the site-directed mutagenesis approach, it was found that the acidic residues E654, E702, E705, E734, and D738 group together to form two calcium ion-binding sites and, thus, regulate the opening of the channel [22,23] (Figure 1). Each monomeric subunit is regulated independently [24,25]. The N-terminal domain also participates in an intra-subunit interaction with the cytoplasmic C terminus, the Cα1 helix. In this proposed cryo-EM structure, the channel is supposed to be nonconducting because ANO1 reduces calcium in a time-dependent manner, pushing the channel into an inactive state [20,26]. Despite several attempts by researchers to decipher the structure of ANO1 in the open state, the fully open pore structure of ANO1 has not been elucidated [27].

### 2.2. Regulation and Activation of ANO1

Most of the natively expressed CaCCs are sensitive to cytosolic calcium in very low concentrations (100–600 nM), as well as membrane potential [28,29,30]. The CaCCs expressed in many tissues show synergistic activation in response to intracellular calcium and voltage [31,32,33]. The synergistic regulatory mechanism is due to the calcium-binding site localized within the transmembrane segment of the channel, which makes CaCCs sensitive to membrane potential [33,34,35]. A similar regulatory mechanism has been reported with ANO1, as it is also sensitive to both calcium and voltage. According to a few studies, the required calcium concentration for the activation of ANO1 ranges from 0.4–0.6 µM at a positive membrane potential, and this value increases as the membrane potential deviates to the negative side [16,36,37]. Studies have also shown that ANO1 is activated in the presence of other divalent ions such as Ba^2+^, Sr^2+^, and Ni^2+^, along with calcium [38,39,40]. The molecular mechanism responsible for the channel’s gating under the influence of calcium and voltage is unknown. However, experiments in isolated membranes have shown direct activation of ANO1 in response to increased intracellular calcium concentration [39]. However, the specific protein or mechanism responsible for channel activity’s slow and inevitable breakdown is yet unknown. Notably, studies have shown that this reduction in the activity of isolated membrane ANO1 over time can be prevented in the presence of ATP and calmodulin via an unknown mechanism [41,42]. Concomitantly, ANO1 conductivity was significantly reduced in the presence of calmodulin inhibitor trifluoperazine or inhibitory peptides [41]. However, contrary to this finding, Terashima et al. (2013) did not find any modulation in the activity of the ANO1 channel in the presence of calmodulin and proposed that calmodulin may not be essentially required for the activation of ANO1 [33]. Overall, these contrary findings indicate that further investigation is required to elucidate the exact role of calmodulin in the activation of ANO1.

Phosphorylation is another possible mechanism known to be involved in the regulation of ANO1 [42]. The potential phosphorylation sites in ANO1 have already been identified in the analysis of ANO1 amino-acid sequencing [43] (Figure 1). However, studies have shown that phosphorylation does not always have a stimulatory effect on ANO1, and contrary results have also been reported [44]. Hence, inclusive studies are required to trace the specific phosphorylation site and how this modulates channel activity in an in vivo environment.

Phospholipid phosphatidylinositol-(4,5)-bisphosphate (PIP_2_)-mediated regulation of ANO1 has also been reported in the literature [45,46]. Studies have shown that decreased PIP_2_ in rat PASMCs has a negative impact on the activation of ANO1. However, Ta et al. showed a significant increase in channel activity in HEK-293 expressing ANO1 when treated with diC8-PIP_2_ [47]. Earlier studies have established that the activation of cells with phospholipase C causes an increase in the production of IP_3_, which in turn triggers the release of calcium from intracellular space and may activate ANO1. Subsequently, the release of intracellular calcium delays the release of IP_4_; thus, a similar model may work as an inhibitory feedback mechanism of ANO1 [48]. Some fatty acids such as stearic, arachidonic, oleic, and docosahexaenoic acid were also found to have inhibitory effects on ANO1 activity in a concentration- and voltage-dependent manner, probably due to altered lipid channel–protein interaction [49].

### 2.3. Expression of ANO1

ANO1 is ubiquitously expressed in all secretory exocrine glands in the epithelia (salivary gland, mammary gland, pancreas, kidneys, and gut), notably in airway epithelial cells [50]. In humans, ANO1 is expressed throughout the respiratory tract from the large bronchi to the alveoli [51]. Moreover, ANO1 is present in interstitial Cajal cells that generate the pacemaker activity of gastrointestinal smooth muscles [52,53], as well as in smooth muscles of the respiratory and reproductive systems [54] and sensory neurons. It is also expressed in proliferating cells of different types [55].

### 2.4. Physiological and Physiopathological Functions of ANO1

**Figure 2:**
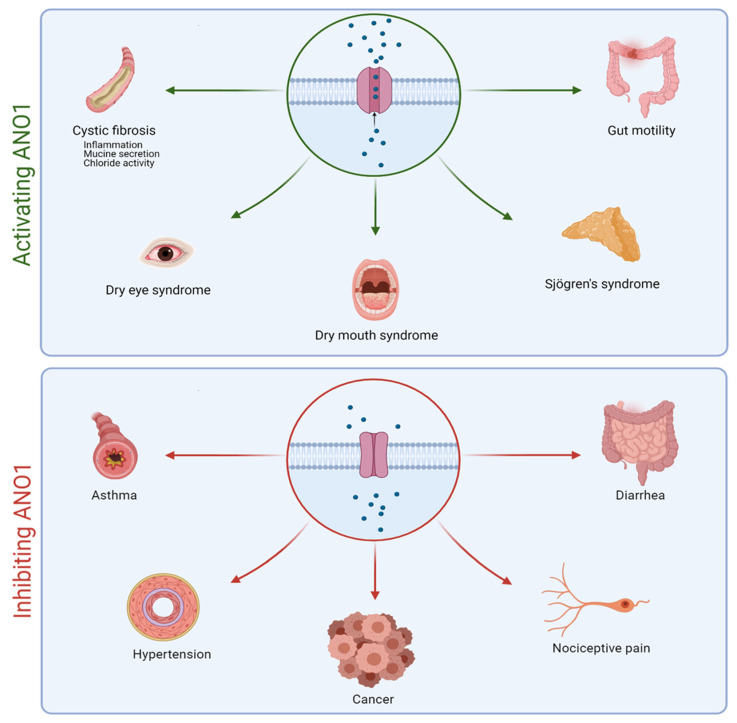
Schematic representation of the positive impacts of activating or inhibiting ANO1 in different pathologies.

Due to its presence in many cell types, the ANO1 channel is involved in several important physiological and pathophysiological processes such as the regulation of smooth muscle contraction, nociception, and fluid secretion in the salivary glands [50,56] (Figure 2). ANO1 hyperactivity plays a role in asthma and neuropathic pain, while it is also involved in cell migration and proliferation in different cancers [57,58,59,60,61,62]. Ruffin et al. demonstrated a significant decrease in cell proliferation and migration when ANO1 is decreased [63]. In contrast, a decrease in ANO1 activity is associated with dry eye and dry mouth syndromes, Sjögren syndrome, and CF (Figure 2).

Rock et al. showed the importance of ANO1 in the development of the murine trachea [64]. All homozygous mice for the ANO1 null allele died within 1 month of birth and exhibited severe tracheomalacia with gaps in the tracheal cartilage rings along the entire length of the trachea, as well as a mucus build-up leading to airway obstruction and contributing to neonatal mortality [64,65]. Impaired mucociliary clearance has also been demonstrated in ANO1 KO mice, suggesting that ANO1 could participate in mucociliary clearance regulation in the airways [66].

One study showed that ANO1 overexpression decreases proinflammatory cytokine interleukin-8 (IL-8) secretion in CF bronchial epithelial cells [67], while a different study showed that ANO1 is upregulated in inflammatory conditions and is associated with airway goblet cell metaplasia [68]. Huang et al. showed that ANO1 is specifically expressed in mucus cells and poorly expressed in ciliated cells. The induction of mucus cell hyperplasia with IL-4, a potent inducer of mucus secretion and mucus cell metaplasia in the airways, increases the number of cells co-expressing ANO1 and mucin 5AC (MUC5AC, predominant mucin produced by airway epithelial cells) [69]. Scudieri et al. suggested that ANO1 expression is particularly required under mucus hypersecretion conditions to ensure adequate water and electrolyte secretion [68].

ANO1 is also involved in bicarbonate ion secretion following the direct association of ANO1 and calmodulin at high cytosolic calcium concentrations [70].

## 3. ANO1 in Cystic Fibrosis

### 3.1. Overview

A study showed that the activity of CaCCs, in general, was increased in the nasal epithelium in vivo of patients with CF [71]. In 1986, another team demonstrated the presence of chloride current at the apical membrane of epithelial cells of CF in the presence of ionized calcium [72]. Moreover, another study showed a decrease in ATP-induced chloride efflux in the primary bronchial epithelial cell line [73]. The identification of ANO1 as a CaCC later on and its involvement in many deregulated processes in patients with CF made it a real therapeutic target. In the last few years, more research has been dedicated to ANO1 in CF.

**Figure 3:**
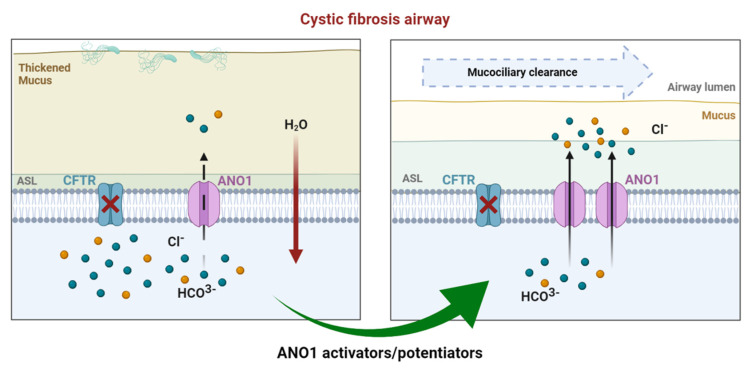
In CF airways, dysfunctional CFTR leads to compromised chloride efflux. Sodium entry is upregulated, leading to a dehydrated air surface liquid (ASL) and impaired mucociliary clearance favoring mucostasis, causing chronic inflammation and infection. In healthy airways, ANO1 is colocalized with CFTR within the apical membrane of epithelial cells, contributing to ion and water homeostasis. In CF ciliated cells, the expression of ANO1 is also diminished. Modulating ANO1, as an alternative CF therapy, could compensate for defective CFTR and, thus, enhance fluid secretion by ciliated epithelial cells, regulating ASL height and pH.

Recent data reported the absence of calcium-induced chloride currents in epithelial cells of ANO1 KO mice [74]. A subsequent study showed that CFTR expression requires ANO1 in plasma membranes, indicating a close relationship between the two chloride channels [75]. Moreover, ANO1 expression and chloride activity were decreased in CF [63]. According to several observations ((1) ANO1’s absence decreases airway secretion, (2) ANO1 is ubiquitously expressed in all the tissue affected by CF, including airway epithelial cells where ANO1 is a secondary chloride channel, (3) ANO1 provides a chloride pathway that is CFTR-independent, and (4) ANO1 is involved in HCO_3_^−^ secretion, which is highly important for fluid secretion and mucus hydration [76]), increasing ANO1 activity can probably compensate for CFTR deficiency (Figure 3). Different approaches targeting ANO1 have been developed to bypass CFTR dysfunction.

### 3.2. Drug Approaches Targeting ANO1 in Cystic Fibrosis

Many molecules have been used to modulate ANO1’s activity, but few were shown to be efficient and, most importantly, specific to ANO1 (Table 1).

Long before discovering ANO1’s function in the airways, a clinical trial targeting CaCCs, in general, took place. Inspire Pharmaceuticals developed denufosol (INS37217), the first CFTR-independent drug for CF lung therapy, carried into clinical trials in 2001 [77,78,79,80]. The inhaled P2Y2 receptor agonist activated P2Y receptors and led to intracellular calcium activation of chloride efflux through CACCs. This increased airway epithelial chloride efflux in vitro, increasing airway fluid volume. However, the second phase III clinical trial of denufosol was disappointing as it did not achieve statistical significance for its primary efficacy endpoint in improving forced expiratory volume in 1 s (FEV1). This failure was due to (1) denufosol targeting all CaCCs, (2) its short half-life in vivo due to rapid degradation by ectonucleotidases, and (3) increased intracellular calcium stimulating goblet cell exocytosis that might have led to an increase in mucus in the airways. Therefore, CaCC activators need to target CaCCs directly without elevating cytoplasmic calcium for more targeted therapy and efficacy [81].

ANO1 identification paved the way for the development of specific activator molecules which would, without modifying the calcium signaling, obtain a more sustained activation over time, leading to better efficiency.

Another novel drug is an ANO1 potentiator (ETD002) developed by Enterprise Therapeutics (based in the University of Sussex Innovation Center, UK) and acquired by Roche (Genentech) in October 2020. This inhaled molecule demonstrated an upregulation of ANO1, which boosts epithelial fluid secretion and mucus clearance in primary CF bronchial epithelial cells and ovine models. Unlike denufosol, intracellular calcium measurements checked that ETD002 did not affect calcium mobilization, coherent with a direct effect on ANO1. A phase I study to test the safety of ETD002 in healthy participants is in progress [82]. The mechanism via which ETD002 potentiates ANO1 activity is still unclear.

Our team has also worked on an innovative alternative approach using a locked nucleic acid (LNA)-enhanced antisense oligonucleotide (ASO). A previous study demonstrated that microRNA (miR-9) contributes to the downregulation of ANO1 expression and activity by directly targeting its 3’UTR. ASO ANO1 binds to the 3’UTR target site of ANO1 mRNA, preventing miR-9 from gaining access to that site [83]. ASO ANO1 has increased ANO1 expression, chloride activity, and mucus clearance in primary human CF cells and CF mice. Recent studies have suggested that ANO1 plays an essential role in mucus production [84]. To date, we have not observed any significant increase in mucin 5AC (MUC5AC, the main component of respiratory mucus produced by goblet cells) or mucin 5B (MUC5B, gel-forming mucin that plays a key role in mucociliary clearance) expression in vitro.

Currently, ANO1 activation as a therapeutic target is subject to controversial opinions. Centeio et al. found an upregulation of ANO1 expression in submucosal glands, airway smooth muscles, and pulmonary blood vessels in CF and asthmatic inflamed lungs [105]. Moreover, activating ANO1 with Eact, which also activates other channels, induced mucus production in airway goblet cells and bronchoconstriction in ovalbumin-sensitized asthmatic mice [85], whereby activating ANO1 could worsen the pathology in inflammatory airway diseases. Instead, the authors demonstrated that ANO1 inhibition by niclosamide significantly reduced goblet cell metaplasia and mucus production in asthmatic mice, as well as inhibited MUC5AC expression in Calu-3 human submucosal cells, suggesting that ANO1 inhibition might be beneficial in inflammatory airway diseases [105]. It is also important to note that a transcriptome meta-analysis revealed that CF and asthma pathways are highly divergent [106]. Furthermore, the same group reported a defect of mucus secretion and accumulation in secretory cells in 2018 when ANO1 was knocked out in ciliated airway epithelial cells and intestinal goblet cells, highlighting the vital role of ANO1 in mucus secretion [84]. Another study showed that ANO1 inhibitors reduced both mucus secretion and airway hyperactivity [69].

On the other hand, using a human respiratory basal cell line (BCi-NS1.1), Simões et al. showed that MUC5AC production does not require ANO1, and their simultaneous upregulation is only circumstantial under cell proliferation [107]. The authors also showed a decrease in ASL when inhibiting ANO1. Furthermore, while replying to Olschewski et al.’s concerns on increasing ANO1 activity, Danahay et al. declared that positive modulation of ANO1 induces no bronchospasm in the conscious sheep model nor affects vascular smooth muscle contraction (unpublished observations) [108,109]. Overall, ANO1’s possible role in mucus production remains obscure and evokes controversial opinions over the beneficial or deleterious results of stimulating the channel in CF.

It is critical to emphasize the importance of this CFTR-independent strategy regarding the 10–15% of struggling patients, notably class I patients and nonresponders, who cannot benefit from Vertex’s molecules and should not be forgotten. This strategy might also be combined with CFTR modulators for additive/synergistic actions to enhance clinical outcomes.

In a recent case report, seven male patients between 17 and 39 years of age, treated with Trikafta^®^, reported testicular pain [110]. It is suggested that this may be related to CFTR function restoration in the male reproductive tract. In the same manner, we can probably expect not only an ANO1 activation in the airways but also in multiple tissues affected by CF and in which ANO1 is expressed, such as the male reproductive tracts, the liver, and gallbladder, the proximal digestive tract, and the gastrointestinal tract (https://www.proteinatlas.org; accessed on October 2021). Hence, patients with CF can be treated on multiple fronts by using an alternative non-CFTR chloride pathway to compensate for defective CFTR. Moreover, ANO1 might help repair and regenerate CF airways because ANO1 upregulation is correlated with enhanced proliferation [63]. Furthermore, relieving gastrointestinal (GI) symptoms in patients with CF was set as a “top 10” priority by the CF community [111]. Since the role of ANO1 in gut motility has been demonstrated in mice homozygous for the null ANO1 allele [112,113], we hypothesize that activating ANO1 might have a therapeutic effect in patients with CF who suffer from intestinal obstruction syndromes. Hence, improving their nutritional status is also associated with better clinical outcomes [114].

## 4. Conclusions

Although there are still some reservations concerning ANO1’s role in mucus production, enhancing ANO1’s activity constitutes an up-and-coming alternative for treating patients with CF independently of CFTR mutations, alone or in combination with CFTR potentiators and correctors for better clinical efficacy.

## Figures and Tables

**Table 1:** Summary of ANO1 inhibitors and activators used in CF.

Inhibitors	Specificity	Assay	References
ANI9	Not specific	In vitro	[82,85]
CCinh-A01	Not specific	In vitro, in vivo	[86]
DIDS	Not specific	In vitro	[87]
Diphenylamine-2-carboxylate (DPC), 5-nitro-2-(3-phenylpropylamino) benzoic acid	Not specific	In vitro	[88]
Flufenamic acid	Not specific	In vitro	[89,90]
Niclosamide	Not specific	In vitro, in vivo	[91]
Niflumic acid	Not specific	In vitro, in vivo	[92]
Plumbagin	Not specific	In vitro	[93]
Quercetin	Not specific	In vitro, in vivo, clinical trial (phase II)	[94,95,96]
Tannic acid	Not specific	In vitro	[97]
T16ainh-A01	Specific	In vitro	[86]
**Activators**			
Denufosol (INS37217)	Not specific	In vitro, in vivo, clinical trial (phase III failed)	[77,78,79,80,81,98,99]
Duramycine (MOLI1901)	Not specific	In vitro, in vivo, clinical trial (phase II failed)	[100]
Eact	Not specific	In vitro	[86]
ETD002 (or ETX001)	Specific	In vitro, in vivo, clinical trial (phase I)	[101]
Interleukin 4	Not specific	In vitro	[14]
Resveratrol	Not specific	In vitro, in vivo, clinical trial	[102,103,104]
TSB ANO1	Specific	In vitro, in vivo, preclinical	[83]
